# Time-Lapse Observation of Crevice Corrosion in Grade 2205 Duplex Stainless Steel

**DOI:** 10.3390/ma16155300

**Published:** 2023-07-27

**Authors:** So Aoki, Dirk L. Engelberg

**Affiliations:** 1 Nuclear Science and Engineering Center, Japan Atomic Energy Agency, 2-4 Shirakata, Tokai-mura, Naka-gun, Ibaraki 319-1195, Japan; 2 Metallurgy & Corrosion, Department of Materials, The University of Manchester, Oxford Road, Manchester M13 9PL, UK

**Keywords:** stainless steel, polarization, potentio-static, selective dissolution, crevice corrosion, gas evolution

## Abstract

The objective of this study was to investigate and visualize the initiation and propagation of crevice corrosion in grade 2205 duplex stainless steel by means of time-lapse imaging. Transparent Poly-Methyl-Meth-Acrylate washer and disk were coupled with duplex stainless steel to create an artificial crevice, with electrochemical monitoring applied to obtain information about the nucleation and propagation characteristics. All nucleation sites and corroding areas inside crevices were recorded in situ using a digital microscope set-up. Localized corrosion initiated close to the edge of the washer, where the crevice gap was very tight, with active corrosion sites then propagating underneath the disk into areas with wider gaps, towards the crevice mouth. The growth was associated with a rise in anodic current interlaced with sudden current drops, with parallel hydrogen gas evolution also observed within the crevice. The current drops were associated with a sudden change in growth direction, and once corrosion reached the crevice mouth, the propagation continued circumferentially and in depth. This allowed different corrosion regions to develop, showing selective dissolution of austenite, a region with dissolution of both phases, followed by a region where only ferrite dissolved. The effect of applied electrochemical potential, combined with time-lapse imaging, provides a powerful tool for in situ corrosion studies.

## 1. Introduction

Duplex stainless steels (DSS) have a dual-phase structure consisting of an austenitic (γ) phase present in an island-like pattern within the ferritic (α) matrix phase. Compared to standard austenitic stainless steels such as Type 304 and Type 316, DSS has a lower Ni content and a higher Cr and Mo content. In general, DSS are superior in mechanical properties such as proof stress and tensile strength and corrosion resistance against localized corrosion such as pitting corrosion, crevice corrosion and stress corrosion cracking (SCC) to general purpose austenitic stainless steels or ferritic stainless steels because of their chemical composition and their dual phase structure. Therefore, DSS are used in severe corrosive environments like high chloride concentration solutions [1,2,3] or oil and gas critical infrastructure. However, it has been reported that DSS can develop crevice corrosion when exposed to seawater [4,5,6]. The crevice corrosion behavior and its mechanism in DSS have not been fully understood yet, with a major challenge related to the corrosion of both phases present in DSS.

Crevice corrosion can occur in relatively mildly corrosive environments, and in engineering structures consisting of a number of components, crevice sites are unavoidable. Once crevice corrosion is initiated, the rate of propagation can be quite fast [6,7], with increased crevice corrosion rates reported under stress [8,9]. It has also been reported, in general, that crevice and pitting corrosion are accelerated in the presence of mechanical stresses [10,11]. Accordingly, the occurrence of crevice corrosion is still a major concern for the application of duplex stainless steel in severe service environments, and it is of pragmatic importance to develop effective measures toward its prevention based on the underlying corrosion mechanism.

Yakuwa et al. [5] distinguished three distinct regions within a crevice, from the fringe to the center, when DSS is exposed to natural sea water in the Red Sea and the Arabian Gulf for over one year. This result showed that preferential dissolution of either the α-or γ-phase can proceed within the crevice of a DSS. The author has [12,13] systematically investigated the influence of the potential on the preferential dissolution behavior of both the α-or γ-phases in DSS in high chloride concentration and low pH solutions. In addition, there are several available reports on corrosion behavior for several DSS’s in varying environments [14,15,16,17,18,19,20,21,22,23,24,25].

Crevice corrosion with preferential dissolution of duplex stainless steels has also been reported by J. N. Al-Khamis et al. [26] using the IR mechanism. However, this study was carried out in an acid-chloride solution environment and therefore analyzed the corrosion behavior after sufficient growth of crevice corrosion.

Recently, various in situ observation techniques have been used to obtain a more in-depth understanding of corrosion kinetics. For example, direct observation of atmospheric corrosion rates of duplex stainless steels by X-ray tomography over approximately two years has been reported, where localized corrosion and preferential dissolution on DSS were observed, in situ [27].

The research reported here is centered on providing supporting evidence of the nucleation and propagation characteristics of crevice corrosion in 2205 DSS in a neutral chloride environment, with a focus on better understanding the dissolution behavior of each phase within the crevice. To elucidate this degradation mechanism, a thorough understanding of the spatial and temporal propagation of corrosion is necessary.

The objective of this study was to investigate the crevice corrosion initiation and propagation behavior of grade 2205 duplex stainless steels by means of in situ imaging observations using a transparent washer-crevice set-up.

## 2. Materials and Methods

A type 2205 (UNS S32205) DSS sheet with the chemical composition shown in Table 1 was used for this study.

**Figure 1:**
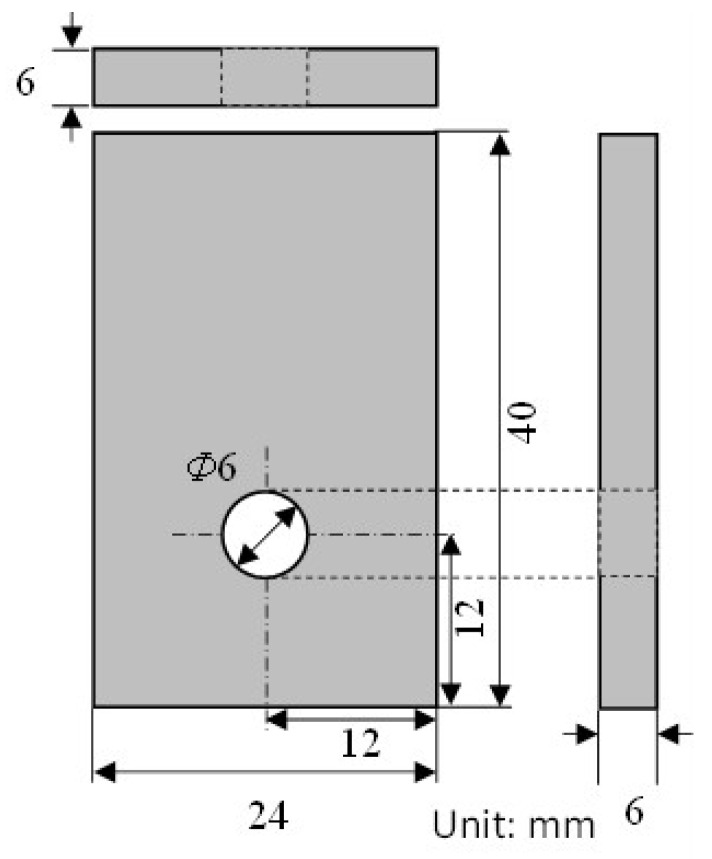
Schematic drawing of test piece for crevice corrosion test.

The test piece dimensions were 40 × 24 × 6 mm (L × W × T mm). A hole with ø6 mm in diameter was drilled into the sample, with Figure 1 showing a schematic drawing of the rectangular test piece.

The entire surface was wet polished with #600 SiC grinding paper to remove any contaminants and create a uniform and controlled surface condition for subsequent crevice corrosion tests. The electrolyte used was a reagent-grade 0.6 M NaCl solution with a constant test temperature of 50 ± 1 °C.

**Figure 2:**
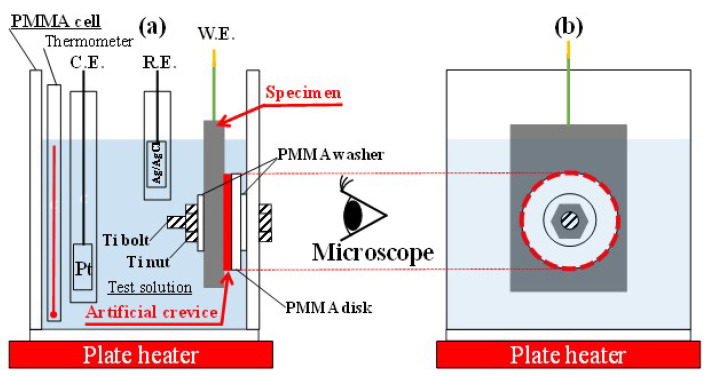
Schematic illustration of the specimen arrangement for the in situ observation ((**a**) cross section, (**b**) front) (PMMA: poly-methyl-methacrylate, W.E.: working electrode, R.E.: reference electrode, C.E.: counter electrode).

For all electrochemical measurements, a Pt counter electrode and an Ag/AgCl reference electrode (KCl sat.) were used. In the following text, all potential values are expressed in V vs. Ag/AgCl. For carrying out in situ observation of both crevice corrosion initiation and propagating behavior, the DSS test piece was interfaced with transparent poly-methyl-methacrylate (PMMA) artificial crevice formers, made up of a washer and a disk. A transparent PMMA was chosen to be able to observe the local dissolution behavior on the test piece surface by means of an optical microscope. The observed dissolution behavior was then correlated to the recorded current trace over time to understand the development of the corroded crevice sites. Figure 2 shows the schematic configuration of the in situ observation system, which was held together with electrically isolated Ti bolts.

The artificial crevice-forming PMMA disk (ø20 mm) and PMMA washer (ø12 mm) were inserted between the test piece and the PMMA electrolytic cell wall, with in situ observations carried out through the outside sidewall of this PMMA cell set-up.

The surface for the in situ observation was further wet polished with SiC up to a #1200 finish, just before the crevice corrosion set-up was assembled. Thereafter, the PMMA disc and washer were set between the test piece and the PMMA electrolytic cell wall by tightening the Ti bolt and nut with a torque of 1.96 Nm, with the sample immersed in the test solution. This ensured that electrolyte was able to remain inside the crevice, here simulated between the disk and sample surface. In this configuration, a constant pressure was applied to the crevice zone beneath the PMMA washer, while with increasing radial distance from the center of the crevice, the crevice gap tended to increase due to the nature of the PMMA cell set-up [28].

The open circuit potential (OCP) was measured, and the sample was then potentio-statically polarized with the in situ observation test. Applied potentio-static conditions were +0.01 V, +0.03 V, and +0.05 V vs. Ag/AgCl. Three potentio-static tests were carried out, with a new sample used for each individual test. The chosen potentials are above the threshold potential for crevice corrosion initiation of the specimen at this exposure temperature, since the aim was to observe both nucleation and growth of a crevice. The critical pitting corrosion temperature and associated Pitting Resistance Equivalent Number (PREN), which gives an indication of the temperature range for initiating crevice corrosion, can be estimated using the chemical composition [29,30]. The DSS composition here gives an estimated PREN of 38.4 and a CPT of 45 °C. This means that if the potential of the specimen is held at these chosen potentials, crevice corrosion can be expected to occur. In addition, in order to explore the influence of holding potentials on crevice corrosion behavior in this study, three potential conditions were used, varying by 0.02 V.

The current response of the test piece was recorded over time. The corroding area inside the crevice was continuously monitored, and an image was taken with the microscope at 15 s intervals during the test. The total duration for each test was 120 min, resulting in 481 images taken during each test, including the reference condition at t = 0 s. The dissolved area over time was analyzed with ImageJ analysis software, using a time interval of 16 images, resulting in a snap shot every 4 min [31]. After importing the images into ImageJ and setting the scale, the edges of the corroded area, i.e., the corroded/base metal boundary, were edged with a polygon section, and the area of the corroded area was measured. The height profile of the tip of the corroded area after the test was completed was measured using a wide-area 3D profilometer, the Keyence VR-3200 (Keyence, Tokyo, Japan). Furthermore, for the test at +0.03 V, the corroded area inside the crevice after the test was observed using a scanning electron microscope (SEM). In order to understand the preferential dissolution in the corroded area, energy dispersive x-ray (EDX) analysis was carried out to identify the ferritic (α) and austenitic (γ) phases based on their chemical composition. The elements used for discrimination were Cr, Ni, and Mo, which are effective elements for corrosion resistance and typically have characteristic concentrations in each phase, with Fe defining the matrix element. These four elements were added together to obtain an approximated value of 100 mass% (Cr + Ni + Mo + Fe = 100 mass%) and used for the discrimination of each phase.

## 3. Results

**Figure 3:**
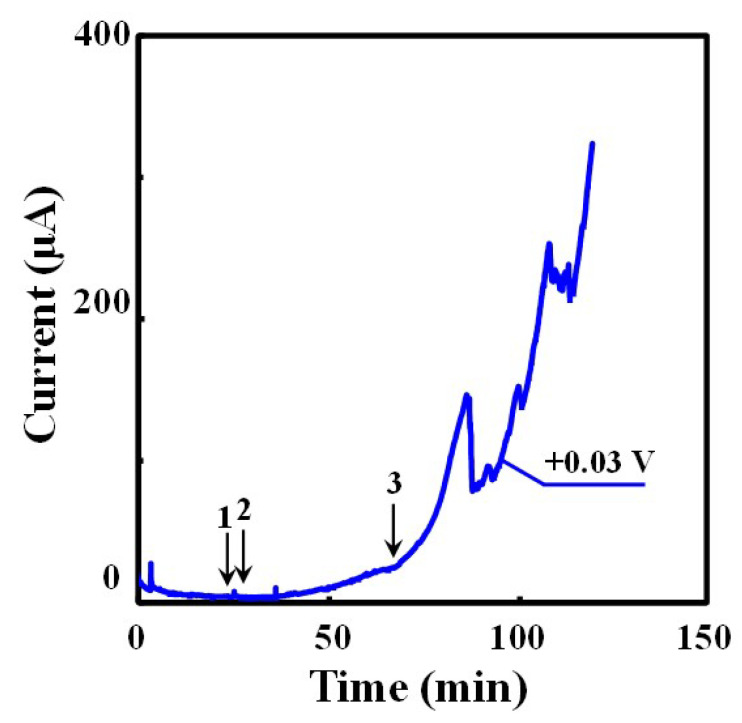
The change of anodic current over test time (Black arrows and numbers indicate initiation of each corrosion site) (UNS S32205, 0.6 M NaCl, 50 ± 1 °C, +0.03 V vs. Ag/AgCl).

The measured OCP of the duplex sample for all three tests ranged between −0.118 V and −0.127 V. The sample was then potentio-statically polarized, and the current versus time was recorded. In parallel, the earliest onset of corrosion nucleation sites was recorded by monitoring the current response and correlating it to the recorded images, allowing inspection and comparison of all the data in detail. Figure 3 shows the typical change of the anodic current measured during the potentio-static holding at +0.03 V.

The arrows in Figure 3 highlight the initiation of different corrosion sites under the crevice former, where corrosion has been visually identified by analyzing the recoded images. At the early stage of the potentio-static test, the current dropped steadily to 4 μA due to passivation of the test piece. The current started rising after the first 30 min, and then continued to steadily increase over time, interlaced with a number of sudden current drops. These current drops were observed in the samples polarized to +0.03 V and +0.05 V, but not clearly in the samples polarized to +0.01 V. Based on analysis of the in situ observation with discrete intervals of 4 min, the initiation of the first crevice corrosion site in Figure 3 was visually observed after 24 min of exposure, with the second nucleation spot appearing 4 min later, followed by a third independent nucleation point after >60 min. All nucleation sites were located under the crevice former, right at the edge of the washer where the pressure to keep the assembly together was applied. This location at the interface between washer, and crevice former may have provided the right crevice gap geometry, a physical location for electrolyte redox coupling, and access to develop chemistry for the crevice to initiate.

**Figure 4:**
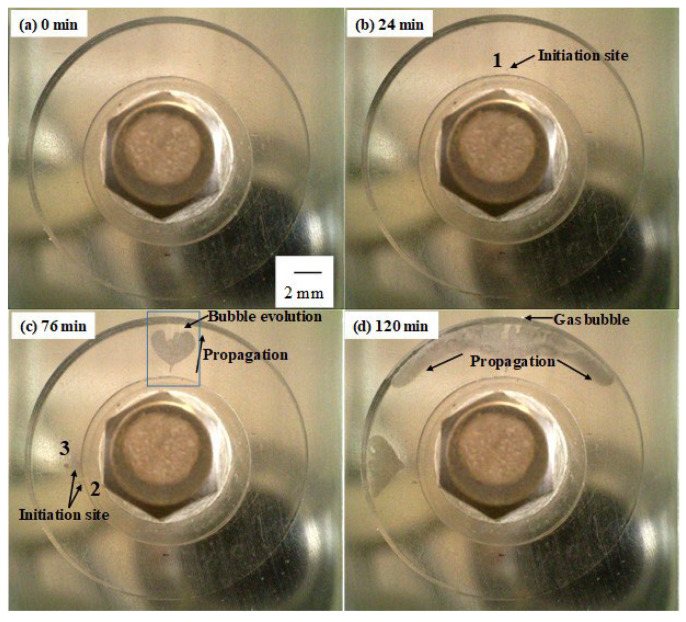
Photographs of the in situ observation of crevice corrosion ((**a**) 0 min, (**b**) 24 min, (**c**) 76 min, (**d**) 120 min) (The numbers correspond to the initiation of each corrosion site in Figure 3) (UNS S32205, 0.6 M NaCl, 50 ± 1 °C, +0.03 V vs. Ag/AgCl).

Figure 4 shows individual snap shots of the in situ observation of the potentio-static crevice corrosion test at +0.03 V, clearly showing the different initiation sites and associated propagation directions.

**Figure 5:**
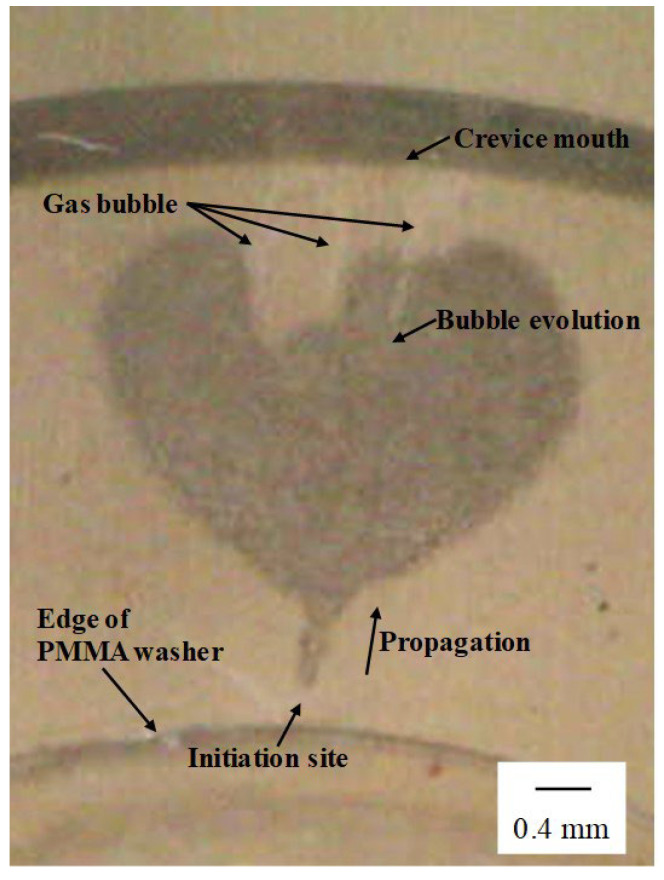
Magnified photograph of crevice corrosion around the area indicated by a square in Figure 4c (76 min) (UNS S32205, 0.6 M NaCl, 50 ± 1 °C, +0.03 V vs. Ag/AgCl).

Figure 5 shows a magnified image of the crevice site in the area outlined by the box in Figure 4c.

As shown in Figure 4b, the first initiation site of crevice corrosion was observed 24 min. after the start of the test, and all corroded areas grew concentrically towards the crevice mouth with time. The drop in current was observed in the +0.03 V polarized sample after approx. 90 and 110 min. (Figure 3), which coincides with the time for both crevices to reach the crevice edge. A large current drop was also observed in the sample polarized to +0.05 V, which was associated with a stop of apparent dissolution at the front, with another part of the area starting to grow from the inside of the crevice to the mouth. These current drops might therefore be related to sudden changes in either the growth directions or even a short time interval of dilution of the crevice environment, with essentially the crevice communicating with the environment outside before the severity of the crevice solution picks-up the growth again. Interestingly, no significant current drop was observed in the +0.01 V polarized sample, possibly because the recorded anodic current was far lower compared to the +0.03 V polarized sample, resulting in a far smaller area of corrosion.

Gas bubble evolution was also observed around the corrosion area, with all bubbles migrating toward the crevice mouth (Figure 4c and Figure 5). There are now two possibilities for gas formation here inside the crevice: either (i) oxygen evolution due to significantly increased acting potential reaching transpassivity and further decomposing the water with oxygen evolution, or (ii) the development of local cathodic sites coupling to and short-circuiting anodic reactions that are close by. The first scenario would mean that the recorded charge is an overestimation of the dissolved crevice volume since the measured anodic current would include both metal dissolution and oxygen evolution. The second scenario with hydrogen evolution would mean that measured charge would be an underestimation of the dissolved volume since a portion of the anodic current is then taken up and compensated by these local cathodic reactions. This description here is, however, a simplification, and the rate of gas evolution would certainly affect the redox reaction rate inside the crevice, providing anodic or cathodic redox active species.

After the crevice reached the crevice mouth, corrosion further propagated circumferentially along the edge of the crevice, growing in size, as shown in Figure 4d. Such results were observed under all test conditions. The crevice gap is very tight at the edge of the washer because of the contact pressure by the Ti bolt, with the crevice gap increasing gradually as it approaches the crevice mouth due to the PMMA disk being slightly warped towards the crevice mouth [24]. The in situ observation suggested that it was not difficult for crevice corrosion to initiate at the site of a tight crevice gap and propagate to grow toward the mouth, where the gap is looser. It is possible that the crevice gap may influence the crevice corrosion behavior if the crevice gap varies with the surface finish or surface morphology of the specimen.

**Figure 6:**
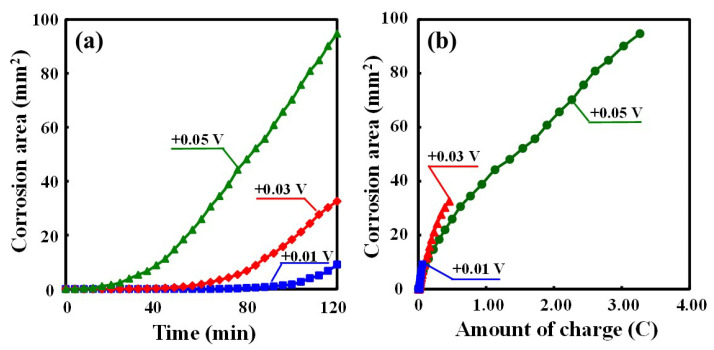
(**a**) The change of the corrosion area over time; (**b**) the correlation between amount of charge and corroded area under every potentio-static test condition (a maximum area under the crevice former of approximately 200 mm^2^) (UNS S32205, 0.6 M NaCl, 50 ± 1 °C).

After the crevice corrosion test was terminated, each image was analyzed to identify the earliest onset of localized corrosion. The corroded area over time as a function of every potentio-static test condition is shown in Figure 6a.

Under higher applied potentials, crevice corrosion initiated earlier, with an increase in corrosion area over time. The time to initiate corrosion from the start of the test was 68 min, 24 min, and 8 min for an applied potential of +0.01 V, +0.03 V, and +0.05 V, respectively. Even at the highest potential of +0.05 V in the test conditions, the corroded area continued to increase with time until the end of the test. The overall corroded area at the end of the test was up to a corroded area of 95 mm^2^ in size, out of a maximum area under the crevice former of approximately 200 mm^2^. A correlation of the measured charge with the corroded area over exposure time for all three different potentio-static test conditions is shown in Figure 6b. In the +0.05 V test, the total amount of charge was, as expected, the highest, with the measured charge scaling with the applied potential.

**Figure 7:**
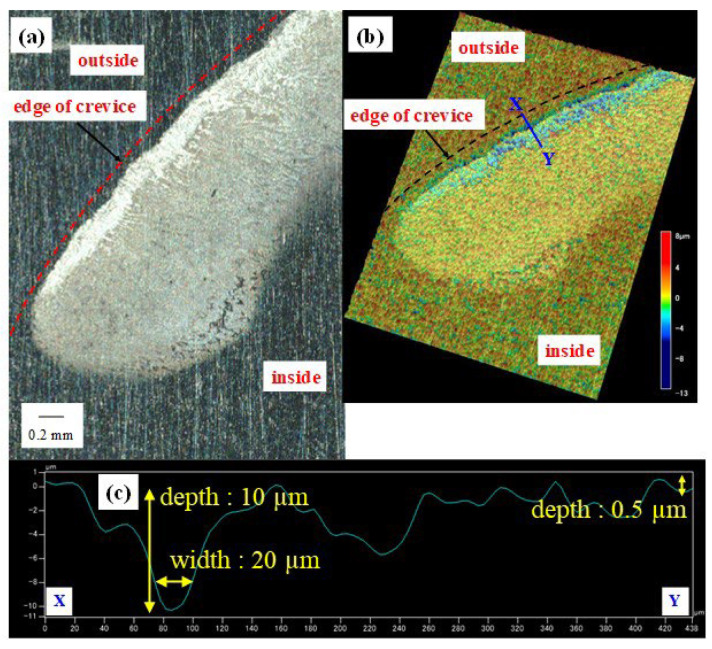
(**a**) Microscopic photograph of the corroded area around the edge of the crevice after the test; (**b**) 3D height distribution image; (**c**) height profile along the X-Y line in (**b**) with the rectangular cross-section value (UNS S32205, 0.6 M NaCl, 50 ± 1 °C, +0.03 V vs. Ag/AgCl).

Figure 7 shows a microscopic image of (a) the corroded area with (b) a 3D height distribution image and (c) the height profile along the X-Y line of the corroded area around the edge of the crevice for the test held at +0.03 V.

The height profile was obtained using a wide-area 3D profilometer, the Keyence VR-3200. As shown in Figure 7c, the depth of crevice corrosion was typically very shallow below 1 µm, with far deeper dissolution observed close to the edge of the crevice former. The deepest dissolution site was approximately 10 µm in depth, located at the outer circle close to the edge. This result indicates that corrosion possibly propagated in a shallow manner until it reached the edge of the crevice former and then spread circumferentially along the edge of the crevice former and grew in parallel in depth. The time to reach the edge from the start of the corrosion test was 108 min, 84 min, and 48 min. for an applied potential of +0.01 V, +0.03 V, and +0.05 V, respectively. A higher applied potential leads to a higher current density and faster growth of the crevice, which clearly shows that the crevice does not grow at the cathodically limiting rate at the applied potential regimes here. The corroded area also grew in depth at the outer edge of the crevice, indicating faster dissolution at these sites compared to all the inner areas, which remained shallow over time.

**Figure 8:**
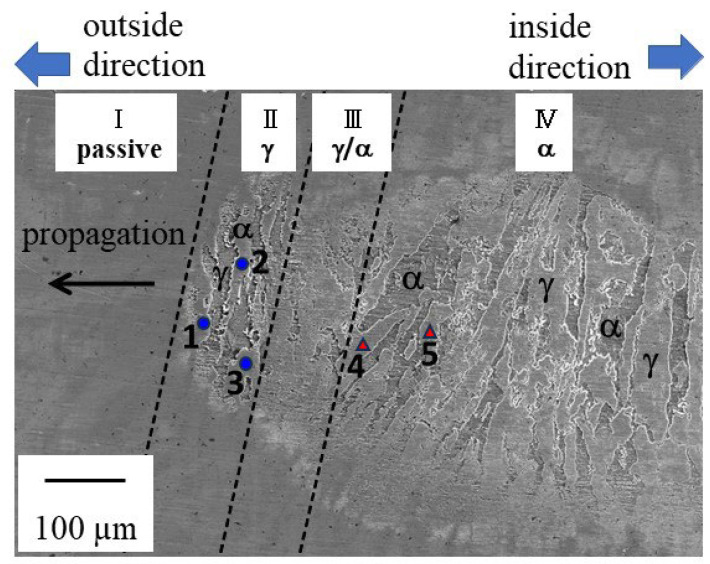
SEM photograph of corroded area inside crevice after the test (I) passive zone, (II) preferential dissolution zone of γ phase, (III) γ phase and α phase dissolution zone, (IV) preferential dissolution zone of α phase (⬤, ▲: EDX analysis point) (UNS S32205, 0.6 M NaCl, 50 ± 1 °C, +0.03 V vs. Ag/AgCl).

A SEM image of the corroded area inside the crevice of the test at +0.03 V is shown in Figure 8.

The left side of this figure shows the outside of the crevice, with the right-hand side showing the center of the crevice. The chemical composition of each of the points shown in Figure 8 is given in Table 2.

The Cr- and Mo-rich phases are the ferritic (α) phases, and the Ni-rich phases are the austenitic (γ) phases. The area around the tip of the corroded site can easily be differentiated into four zones (I-IV) of corrosion behaviors, with the α- and γ-phases in DSS easily distinguished by their chemical composition based on EDX phase analysis. The area around the tip of the corroded site can easily be differentiated into four zones (I-IV) of corrosion behaviors, with the ferritic (α) and austenitic (γ) phases in DSS easily distinguished by their chemical composition. Based on energy dispersive x-ray (EDX) phase analysis, the austenite has higher Ni contents and the ferrite has higher Cr and Mo contents. The area just ahead of the tip (zone I) was not corroded at all, indicating passive material behavior. The zones in the order from the tip of the corroded area towards the center of the sample were labeled as Zone II to Zone IV, with Zone II showing a preferential dissolution zone of the γ-phase, Zone III indicating dissolution of both the γ- and α-phase, followed by a preferential dissolution zone of the α-phase only (Zone IV). This observation indicates that the crevice corrosion mechanism evolves over time, dynamically changing how the microstructure is attacked, possibly related to differences in the acting potential for dissolution.

## 4. Discussion

An interesting and important finding using the in situ observation of the initiation of crevice corrosion is the fact that the corrosion initiation site was already present at the lowest point of the anodic current response curve in Figure 3. The site was already present before a significant current increase was measured. This observation means that crevice corrosion initiated at the lowest passive current of the test piece, and it is not clear whether a pre-cursor already existed at this site. The visual observation method employed was limited to a simple optical inspection.

The volume of corrosion initiation sites underneath the edge of the PMMA washer was all very shallow, possibly supported by the very tight crevice gap underneath the edge of the washer, but it would also appear that the current density at the initiation site was large enough to grow a stable crevice. Because of the small area, the current value is not large and is within the observed passive background current response of the specimen. The local current density must be comparable to active dissolution, but the very small nucleation site resulted in the nucleation not being directly observed by the electrochemical response here. In other words, this means that a small amount of dissolution generates a stable environment for crevice corrosion to grow, as described below. Crevice corrosion propagated to grow towards the crevice mouth, where the crevice gap was wider, resulting in an increasing anodic current response (Figure 3). It has been generally understood that the galvanic cell covering the length from inside the anodic crevice site to the cathodic region outside of the crevice resulted in a large ohmic potential drop (IR drop) with large solution resistance induced because of the tight shape of the crevice [32,33].

In our in situ observation, gas bubble evolution was observed around the corrosion area. As already discussed, these can either be associated with oxygen formation at high anodic potentials or hydrogen evolution at very low potential regions. Here, at the back of the crevice, the potential is far lower than the external holding potential due to the IR drop [34], and the generation of oxygen cannot take place at such a low potential. It is reasonable to assume that hydrogen gas is produced by the local reduction reaction of protons. Pickering et al. also confirmed the generation of bubbles from localized corrosion sites and reported that they were hydrogen gas as a result of mass spectrometric analysis [35]. Metal ions dissolved at the initiation site of crevice corrosion generate protons and therefore decrease solution pH by hydrolysis. Electrons were produced from the anodic reaction of metal dissolution as well as protons. A part of these electrons was then consumed directly on the test piece’s surface inside the crevice, with the local cathodic reaction producing hydrogen gas. This observation suggested that the solution pH in the vicinity of the crevice corrosion area had decreased and that a local cathodic reaction of protons producing hydrogen gas occurred therefore inside the crevice. In principle, it is not possible to directly measure the current based on the electrons consumed by the reaction at the local cathode inside the crevice since the generated cathodic charge is then taken up by local anodic reactions. The effects of cathodic reaction inside crevices should therefore be considered in addition to cathodic reaction on the outside to evaluate crevice corrosion rates. To support this assumption, a comparison was made between the measured volume of material dissolved in the sample polarized to +0.03 V and the charge determined via Faraday’s law from the recorded electrical current vs. time plot. The metal dissolution was assumed to have an average metal cation charge of n = 2.19, an atomic weight of M = 55.79 g/mol, and a density of ρ = 7.87 g/cm^3^, with a Faraday’s constant of F = 96,485 C/mol. The volume of dissolved material was approximated by dividing each corroded site into two discrete areas, with area (1) defined by a rectangular cross-section of the crevice (20 µm width and 10 µm depth), spanning along the circumference, while the other corroded areas inside the crevice were assumed to be 0.5 µm deep. The estimated volume of the corroded area was 20.9 × 10^−3^ mm^3^, and the volume of the corroded area obtained from the measured charge is 15.3 × 10^−3^ mm^3^. This means that the actual volume of the corroded area was approximately 1.35 times larger than the volume of measured anode current. This strongly suggested a contribution of the local cathodic reaction inside the crevice to the actual corrosion rate.

In the correlation between the total charge measured in the corroded area and every potentio-static test condition, the corrosion area increased linearly with increasing charge at each holding potential. Although the corrosion area continued to increase with time until the end of the test, the 3D height distribution analysis revealed that crevice corrosion grew only in depth along the outer edge of the crevice. It was thought that the relationship between amount of charge and corroded area from visual observation deviated from proportionality because crevice corrosion propagated in depth at higher holding potentials. In order for crevice corrosion to continue to grow stably, a high chloride concentration and a low pH solution must be maintained inside the crevice. This means that the hydrolysis reaction of dissolved metal ions and the entry of chloride ions to maintain the charge balance also need to be maintained, which would support the observation of the deeper sites. Therefore, free water, which is consumed in the metal dissolution and its metal ions hydrolysis, needs to be supplied to maintain crevice corrosion spreading circumferentially outside along the crevice. The chloride ions and free water from the outside are likely to be more readily available at the edge than far inside the crevice. For this reason, the crevice corrosion that initiated inside the crevice was expected to propagate in the direction towards the edge of the crevice, and then the crevice corrosion propagated circumferentially along the edge of the crevice. After the crevice corrosion reaches the edge of the crevice, most of the IR drop occurs at the crevice mouth due to the severe crevice thickness. Introducing IR theory from the study by J. N. Al-Khamis et al. [26], the drop from the potential of the passive zone outside the crevice to the corrosion potential of the active peak in the polarization curve was considered to occur at a small distance from the crevice mouth. Likewise, it was thought that the crevice corrosion was able to continue to grow in the depth direction at the edge of the crevice.

The SEM observations after the test showed that the corrosion behavior around the tip of the corroded area was different than the tail towards the specimen center. It has been reported previously that the growth of crevice corrosion maintained four different corrosion morphologies during its propagation stage in DSS [12]. The potential dependence of the preferential dissolution behavior of DSS in a crevice corrosion environment has also been reported [13]. As already mentioned, during crevice corrosion propagation, the outside around the crevice is believed to be cathodic and the inside chiefly anodic. As the mass transfer in and out of the crevice is restricted by the tight gap geometry, the macro-cells in and out of the crevice consist of IR drops due to the large solution resistance. The solution resistance increases with distance from the outer edge of the crevice towards the center, which means that a potential gradient is formed from the outer edge to the center of the crevice. Thus, the potential is noble at the outer edge of the crevice and becomes less noble towards the center of the crevice. This potential gradient is thought to have caused preferential dissolution of the γ-phase in the corroded periphery, followed by dissolution of the α-phase in this zone as the corroded area expanded, and then a change to preferential dissolution of the α-phase as the crevice corrosion propagated. As a result, corrosion grew towards the outer edge of the crevice while maintaining the corrosion morphology of all four regions. The preferential dissolution behavior of the corroded area in the present crevice corrosion test was in good agreement with the findings of other reports (e.g., [36]).

## 5. Conclusions

Crevice corrosion initiation and propagation behavior of Grade 2205 duplex stainless steel were observed by means of in-situ observation and electrochemical monitoring. The crevice corrosion behavior occurring in neutral chloride environments, such as seawater, was visualized and corresponded to the electrochemical response.

Corrosion initiated underneath the edge of the washer, where the crevice gap was very tight, and then corrosion grew concentrically toward the crevice mouth. Under higher applied potentials, crevice corrosion initiated earlier, with an increase in corrosion area over time. A higher applied potential leads to a higher current density and faster growth of the crevice. After corrosion reached the edge of the crevice, it propagated circumferentially along the edge and also in depth.

In situ observation with image analysis revealed that for the nucleation of crevice corrosion, very little anodic current is required before the anodic current increases. This result means that crevice corrosion was initiated by the anodic current response from the passive current of the stainless steel surface.

Gas evolution was observed around the inner dissolved corrosion area. The effects of cathodic reactions within the crevice should be considered for the evaluation of crevice corrosion propagation rates. The measured volume of the corroded area of the sample was estimated at 20.9 × 10^−3^ mm^3^ and the volume of the corroded area obtained from the measured charge is 15.3 × 10^−3^ mm^3^. This means that the actual volume of the corroded area was approximately 1.35 times larger than the volume of measured anode current. This strongly suggested a contribution of the local cathodic reaction inside the crevice to the actual corrosion rate.

## Figures and Tables

**Table 1:** Chemical composition of the specimen (mass%).

Steel	C	Si	Mn	P	S	Cr	Ni	Mo	N
UNS S32205	0.016	0.4	1.5	0.021	0.001	22.4	5.8	3.2	0.18

**Table 2:** Chemical composition of each point shown in Figure 8 analyzed by EDX (mass%) *.

Steel	Cr	Ni	Mo	Fe
point ● 1 (α)	27.45	3.99	4.18	64.38
point ● 2 (α)	27.11	4.51	3.99	64.39
point ● 3 (α)	27.46	4.04	4.72	63.78
point ▲ 4 (γ)	23.76	6.97	2.84	66.43
point ▲ 5 (γ)	23.68	6.98	2.46	66.88

* (Cr + Ni + Mo + Fe = 100 mass%). (●, ▲: EDX analysis point in Figure 8).

## Data Availability

The datasets are available from the corresponding author upon reasonable request.
